# A Deep Morphological Characterization and Comparison of Different Dental Restorative Materials

**DOI:** 10.1155/2017/7346317

**Published:** 2017-06-29

**Authors:** R. Condò, L. Cerroni, G. Pasquantonio, M. Mancini, A. Pecora, A. Convertino, V. Mussi, A. Rinaldi, L. Maiolo

**Affiliations:** ^1^Department of Clinical Sciences and Translational Medicine, University of Rome “Tor Vergata”, Rome, Italy; ^2^Institute for Microelectronics and Microsystems-National Research Council (IMM-CNR), Rome, Italy; ^3^Institute of Complex Systems-National Research Council (ISC-CNR), Rome, Italy; ^4^ENEA, UTTMAT, Casaccia RC, Via Anguillarese 301, 00123 Roma, Italy

## Abstract

Giomer is a relatively new class of restorative material with aesthetics, handling and physical properties of composite resins, and benefits of glass ionomers: high radiopacity, antiplaque effect, fluoride release, and recharge. To verify the superior properties of Giomers, in this study, a deep morphological characterization has been performed with an in vitro comparative study among a Giomer (Beautifil® II by Shofu Dental Corporation, Osaka, Japan), a Compomer (Dyract Extra by Dentsply, Caulk, Germany), glass ionomer cement (Ketac fil plus by 3M ESPE), and a composite resin (Tetric Evoceram by Ivoclar). In particular, mechanical and optical properties and ageing effects have been compared to investigate materials similarities and differences. Indentation tests, UV-Visible spectroscopy, Raman spectroscopy, and weight loss after storage in saliva or sugary drink have been carried out to analyze materials behavior in real conditions. The results confirm the high quality of Giomer material and indicate possible improvements in their usage.

## 1. Introduction

Giomer is a unique class of restorative material. It has been introduced as the true hybridization of glass ionomer (GI) and composite resin and has the distinguishing feature of a stable surface prereacted glass ionomer (S-PRG), which is coated with an ionomer lining incorporated in a resin matrix. This arrangement aids in the protection of the glass core from moisture, adding to long-standing aesthetics, durability, physical and handling properties of composite resins with fluoride release, and recharge property like the GI cement [1–5].

Giomers behave essentially as composite resins; it is doubtful whether the inclusion of S-PRG makes any significant difference to these materials, and they are not fundamentally new materials but a very slightly modified version of well-established materials. Because of their fundamental composite resin nature, from a morphological point of view, Giomer has a better smooth surface than GI and resin-modified glass ionomer cement (RMGIc) and is comparable to composite resin and Compomer, being easier to polish than GIs [6].

Long-term clinical studies have reported satisfactory visual texture and surface roughness of Giomer restorations. Kimyai et al. observed that the use of air-powder polishing device prophylaxis exerted the most detrimental effects on the surface of Giomer, resulting in an increased surface roughness [7].

This phenomenon also occurs after dental bleaching procedures that induce an evident increase in surface roughness both in Giomer and in microfilled composite resin despite the fact that clinically detectable color changes occur in neither of them [8].

It is known that significant surface changes of the dental restorative materials can take place when exposed to low pH drinks for a prolonged period and Giomer is not excluded from this phenomenon. It was shown, in fact, that Giomer suffers a decomposition of resin matrix and fallout of the fillers in composites when exposed to acidic drinks. The exposition to carbonated drink (cola) and orange fruit juice is responsible for the superficial erosion that results in surface roughness. The latter is however less if compared to that which occurs on the surface of Compomer [9].

Kooi et al. have evaluated the effects of food-simulating liquid on roughness and hardness of Giomer and hybrid composite, proving that the hardness of Giomer is most affected by citric acid and ethanol. Therefore, Giomer restoratives were significantly roughened by citric acid [10].

In a recent research, the effects of five beverages (apple cider, orange juice, Coca-Cola, coffee, and beer) on the microhardness and surface characteristic changes of nanohybrid resin composite resin and Giomer have been investigated. After the immersion the microhardness decreased in both materials with a surface degradation that depends upon the exposure time and chemical composition of the restorative materials and beverages [11].

The color stability of Giomer is preserved even after these materials are subjected to ageing in food dyes (food and drink). A study demonstrated this good aesthetic characteristic against various children's beverages (orange juice, Bournvita milk, and coke) and in comparison to RMGIc, Giomer has shown less color changes and a better color stability [12, 13].

Clinical studies demonstrated the excellent aesthetics and clinical stability of Giomer materials. They are employed with success: in class V noncervical lesions restorations of permanent teeth, in class I and II occlusal restorations of posterior primary and permanent teeth, as enamel and protective coatings like pit and fissure sealants, and as a coadjuvant root restorative material in the treatment of gingival recession [3, 6, 14–18].

One of the most important requirements for the success of restoration is related to the prevention of microleakage formation, which is achieved with the proper adherence of restorative material to the cavity walls [19].

Recently Walia et al. have observed that, compared to highly viscous GI cement, zirconia-reinforced, and nanoceramic restorative material, the microleakage is maximum in Giomer, with the lowest sealing ability [20].

As Giomer is a product having cross-linked polymer matrices, the compressive strength and toughness of the material also seem to be higher than the gel network formed by acid-base reaction in glass ionomers. Generally, it is found that the materials having high fluoride release property have low compressive strength. Unlike GIs, Giomer does not have the initial burst of release, and its diffusion-based fluoride release is at a lower level than conventional GIs [2]. However, from clinical demand a material that has high fluoride release and recharge ability as well as high compressive strength is considered a better restorative material. As Giomer is resin-based PRG fillers, its compressive strength is expected to be comparable to any other resin-based material.

Giomers such as the “sculptable” composite are significantly stronger than zirconia-reinforced GI cement, nanoparticle RMGIc, and highly viscous GI cement. Shear strength of the “flowable” injectable hybrid Giomer is intermediate between the composite and GI cement [21].

Quader et al. investigated on compressive strength of four restorative materials: Giomer, composite, Compomer, and GI. By a compressive strength tester, it was possible to observe that the value of compressive strength of Giomer is greater than that of composite and Compomer [22].

Similar results are shown in a study conducted recently by Bollu et al., which shows that Giomer has a poor sealing ability at dentin and cementum margin compared to enamel margins. In terms of marginal adaption it is worse than nanoionomer and RMGIc and presents also the maximum microleakage [23]. As compared to nanoceramic and Ormocer-based restorative material, Giomer holds the worst shear bond strength to dentin together with GI cement [24].

Ilie and Stawarczyk, investigated on the impact of storage up to one year on the micromechanical properties (indentation modulus and Vickers hardness) of dental bioactive restoratives used for bulk-application (Giomer bulk-fill resin composite and GI cement) and their intermediate layer to dentin. No degradation with ageing was identified in the Giomer restorative. The gradual ascending transition in micromechanical properties from dentin through dental bioactive restoratives identified in both restoratives might have a positive effect on the bond quality [25].

In recent years, this new type of glass filler has been receiving attention in clinical papers and on the lecture circuit but still now little published research is available on the properties or performance of Giomers.

Aim of this “in vitro” study was an overall investigation on the morphological and structural characteristics of Giomers in order to make a comparative analysis with three other restorative materials and to provide all information required in terms of processability, mechanical, morphological, and optical properties, crystalline nature, color stability, and hardness. For each of the four materials in exam, the ageing phenomena which can induce a loss in weight or surface changes are observed considering also that many other Giomer products will become available in the future.

## 2. Materials and Methods

In the present in vitro study four different materials in the field of restorative dentistry were examined. In particular we focused on a Giomer (Beautifil II by Shofu Dental Corporation, Osaka, Japan), a Compomer (Dyract Extra by Dentsply, Caulk, Germany), GI cement (Ketac fil plus by 3M ESPE, London, Canada), and a composite resin (Tetric Evoceram by Ivoclar, Amherst, USA).

### 2.1. Specimen Preparation

In order to prepare the samples, a polyurethane stamp was realized with four negative disk specimen shapes (diameter of 4 mm and height of 10 mm) and was placed on a glass slab. Each disc was filled with one of four test materials considered in the study. A transparent matrix strip (Hawe Neos Dental, Bioggio, Switzerland) was placed on the surface of the four restorative materials to produce a smooth and uniform surface in all specimens: a glass slide was then pressed on the stamp containing the restorative materials. Thereafter each material was light-cured from top and bottom using a light source (LED Starlight) at light intensity of 400 mW/cm^2^, with the light tip held perpendicular to the surface of the specimens for 40 sec.

According to this preparation protocol, 4 disk specimens of each of the 4 materials taken in exam were obtained with a total of 16 samples that were divided into 4 randomly groups. For each type of material characterization, the measures of the different groups were analyzed and averaged according to the specific instrumental accuracy.

The samples were stored in distilled water at 37°C.

All the samples were not subjected to a refinishing in order to simulate a clinical situation.

A deep morphological characterization was conducted:

- Mechanical tests: indentation strength test
- Characterization of surface tests: UV-Vis spectrophotometry and micro-Raman spectroscopy analysis
- Ageing test in saliva-comparison
- Ageing test in sugary drink-comparison
- Weight variation test in saliva.

The comparative analyses were performed at the laboratories of the CNR in the Tor Vergata research area.

### 2.2. Indentation Strength Test

The mechanical properties of the different samples were measured by analyzing the imprint obtained from Vickers indentation. In this technique a fixed load (Vickers indenter) is pressed onto the sample and calculating the depth of the area, the imprint caused by the Vickers tip over the samples, the hardness of the material can be calculated:

(1) $$\begin{array}{c} Hv=\frac{\text{Load}\times 1.8544}{\text{Projected Imprint Area}}, \end{array}$$

where Project Imprint Area = diagonal^2^, The load applied for the indentation test was 0.3 Kg.

To determine uniformity of comparison the different samples have been preliminarily subjected to

- three sessions of smoothing process with sandpaper at subsequent grits 1000/2000/5000;
- rinse in alcohol;
- rinse in deionized water;
- drying in nitrogen flux.

Measurements were performed on four samples for each type of resin and the footprint areas on different points of the sample were analyzed. The final value for each material was taken by averaging the measurements.

### 2.3. UV-Vis Spectrophotometry

The optical properties of the samples were studied by measuring the angle-integrated total reflectivity in the spectral range between 200 and 1100 nm with a Lambda 35 UV-Vis spectrophotometer equipped with an integrating sphere. This analysis allows evaluating the response of the four different materials in the visible and near ultraviolet range. Given the noncrystalline nature of the samples, the reflected light was collected through the technique of the integrating sphere.

### 2.4. Micro-Raman Spectroscopy Analysis

Raman spectroscopy analysis was performed to investigate the chemical composition of the samples. The technique is neither invasive nor destructive and provides information on the vibrations of the atoms in a crystal lattice being based on the Raman effect. When a beam of monochromatic light strikes the surface of a sample, several physical phenomena may occur: the radiation can be reflected, transmitted in the material, absorbed, or scattered in all directions. If the wavelength of the scattered light is the same as the incident, one speaks of “elastic or Rayleigh scattering”; if the wavelength is different from the excitation one, it is called “inelastic or Raman diffusion.” In this case, the frequency of the radiation that emerges from the interaction is shifted with respect to the initial one by a quantity equal to that of the lattice vibrations characteristic of the material, so that the spectrum of the scattered light contains fundamental physicochemical information on the investigated sample.

In the study, the measurements were carried out with a Thermo Fisher Scientific DXR Raman Microscope, by fixing the samples on a standard glass slide. A 532 nm laser was powered at 10 mW, and the samples were irradiated with an exposure time of 1 s for 30 accumulations. The spectra were acquired in the range from 50 to 3300 cm^−1^ with a 50x objective. Specifically, the surface of the Raman spectra of the samples of the four different materials with and without contamination has been studied.

### 2.5. Ageing Test in Saliva-Comparison

With the Raman technique any surface modifications of the samples were also studied after ageing for 15 days in human saliva, collected according to the following protocol: the samples of saliva have been collected from 15 healthy male volunteers aged between 25 and 40. Informed consent was obtained from all the volunteers. Participants were asked not to drink, eat, or smoke for at least two hours before the sampling. The saliva has been obtained by using commercial pipettes achieving a volume of 1 ml. The samples have been stored in sterile containers.

Measuring conditions are as follows:

 
*λ* = 532 nm.
 
*P* = 10 mW.
 
Obj = 50x.
 
Exp = 1 s.
 
Acc = 30.

For these four samples the spectra were analyzed and averaged. After removal of the samples from the test tube containing the saliva, a cleaning protocol is adopted which consisted of a rinsing in demineralized and deionized water and alcohol for 5 min, respectively, and drying in nitrogen flow.

Raman analysis has been performed on both sides on the samples in order to evaluate possible differences.

### 2.6. Ageing Test in Sugary Drink-Comparison

The measurements have been performed before and after an ageing of the samples in 30 ml of a sugary drink for 15 days.

 
*λ* = 532 nm.
 
*P* = 10 mW.
 
Obj = 50x.
 
Exp = 1 s.
 
Acc = 30.

Before the test, each sample has been rinsed in deionized water for 5 min and dried in nitrogen to remove possible residues. Different points on the same samples have been collected to observe a map of the surface.

### 2.7. Weight Variation in Saliva

For each type of material, four samples have been immersed in human saliva for increasing time up to 15 days, measuring the weight variation with a precision balance by Mettler Toledo.

According to the same protocol used for the Raman technique, samples were rinsed in deionized water and dried in nitrogen before each measure.

## 3. Results

### 3.1. Indentation Strength Tests

**Figure 1:**
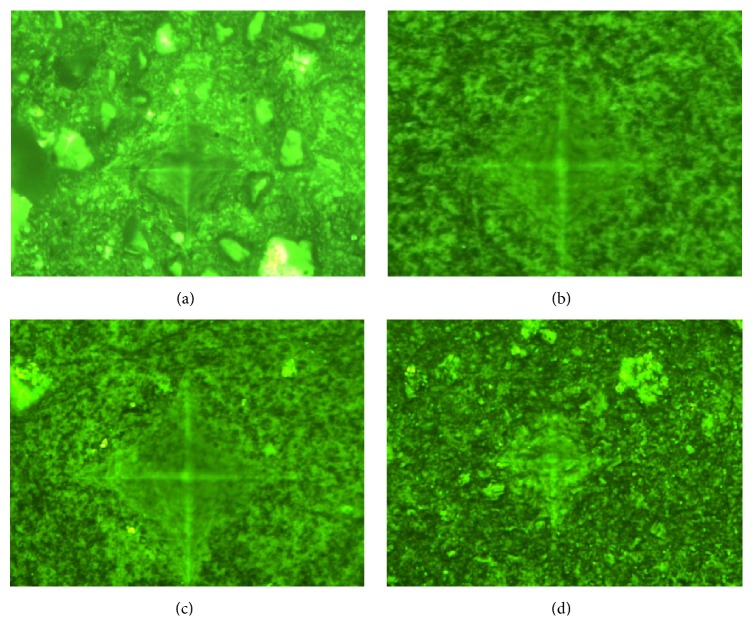
(a) Imprint left for Ketac. (b) Imprint left for Dyract. (c) Imprint left for Tetric. (d) Imprint left for Beautifil II.

In Figures 1(a)–1(d) it is possible to observe an example of an imprint left for each type of material.

The hardness of two materials such as Tetric and Dyract is almost identical as that of Beautifil II is almost double that of the two.

**Figure 2:**
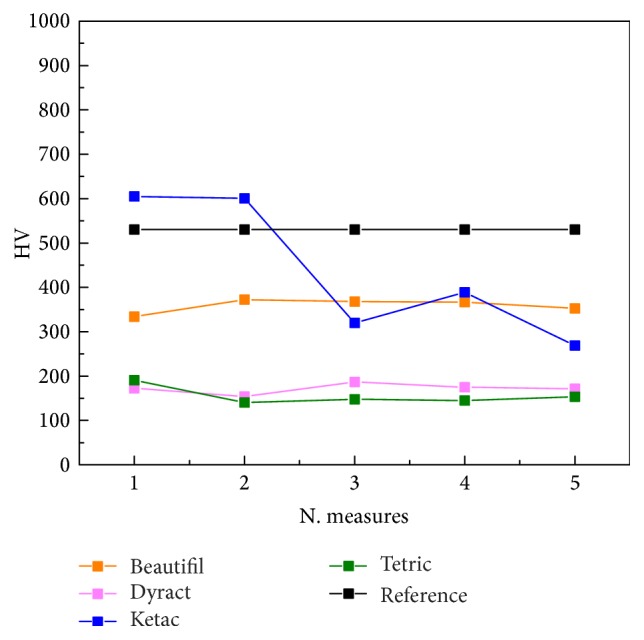
A graph reporting the hardness of the different materials. Prof 2 is a reference while the others are Ketac (prof 1), Tetric (prof 3), Dyract (prof 4), and Beautifil II® (prof 5), respectively.

Ketac shows an even higher hardness, double that presented by Beautifil II, even if there are major fluctuations in the test and in its mapping that would indicate a lack of homogeneity of the hardness of the material in the various sites surveyed. This, however, suggests the presence of two sites basically with hardness 350 as for the case of Beautifil II® and 600 (see Figure 2).

### 3.2. UV-Vis Spectrophotometry

**Figure 3:**
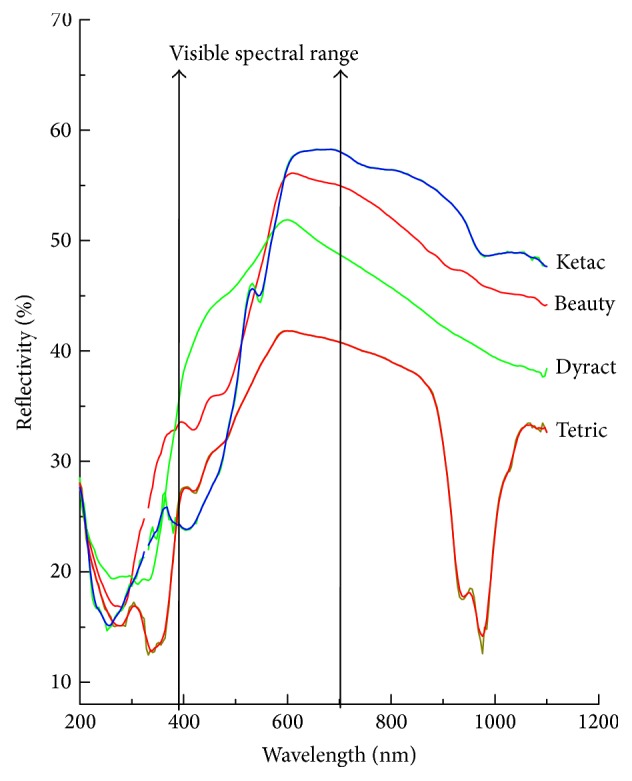
The UV-Vis spectra of the samples.

As shown in Figure 3, where the limits of the visible wavelengths are highlighted, the behavior of all the materials is very similar and there are no peaks of abnormal absorption. Only in the near-infrared does Tetric undergo a significantly different absorption from the other materials. It is also possible to observe a small absorption for Tetric also in the near ultraviolet.

### 3.3. Raman Spectroscopy Analysis

**Figure 4:**
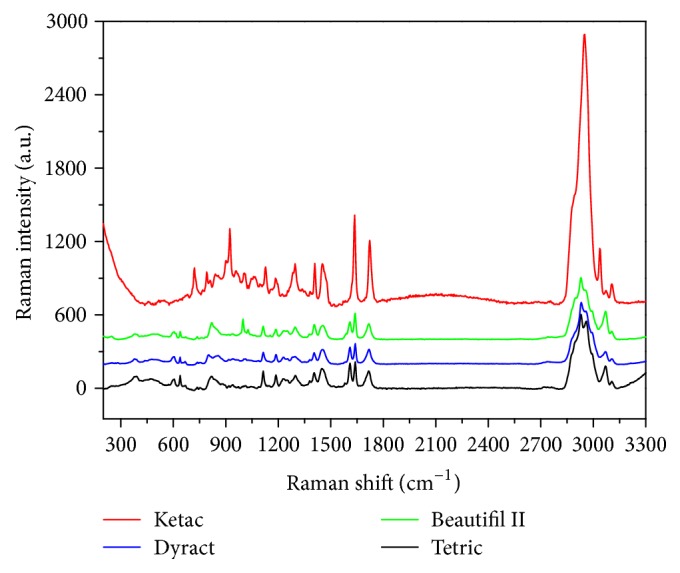
Raman spectra of the four tested materials. The curves have been stacked for clarity.

The Raman spectra of the four materials tested are shown in Figure 4, where they have been stacked for clarity. Even if the curves appear similar, some differences can be noticed, both in the peak intensities and in the number of spectral features, especially in the range from 600 to 1800 cm^−1^, revealing the slightly different chemical composition of the surface layers.

### 3.4. Ageing Test in Saliva-Comparison

After ageing for 15 days in human saliva, any surface modifications of test specimens have been studied with the Raman spectroscopy: it must be pointed out that the samples show different contamination on the two sides. This is probably due to the mucus in saliva condensed on only a side of the sample. Moreover, such surface contamination appears to be of various types and nature, as demonstrated by the spectral differences found on the Raman spectra collected on the two sides of the four samples and presented hereafter.

**Figure 5:**
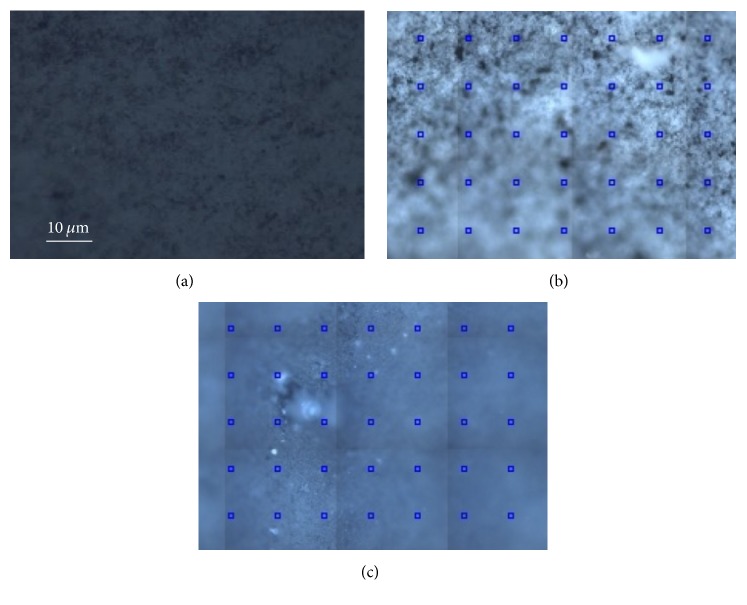
Typical optical images obtained on Beautifil II with a 50x objective. (a) Before contamination; (b) after contamination, front side; (c) after contamination, back side.

As regards Beautifil II, Figure 5 presents optical images collected with a 50x objective before contamination (a) and after the contact with saliva and on the front (b) and back side (c) of the sample (the blue squares represent the different points in which Raman spectra have been collected). More marked differences can be noted between the noncontaminated sample and its front side after exposure to saliva, while the back surface appears less altered by the contamination.

**Figure 6:**
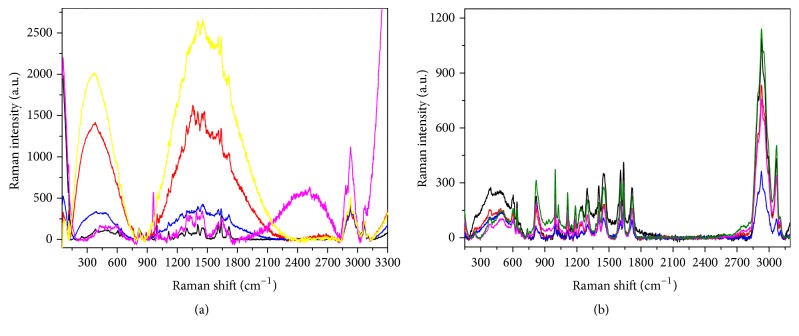
Typical Raman spectra collected on the Beautifil II after contamination. (a) Front side; (b) back side. In both cases the differently colored curves correspond to accumulated measurements taken at some representative points of the grids visible in Figures 5(b) and 5(c).

Indeed, as shown in Figure 6(a), which presents several colored curves corresponding to accumulated measurements taken at different points in the grid visible in Figure 5(b), the Raman spectrum collected on the contaminated front side is not homogeneous on the surface and shows pronounced new broad bands with respect to that realized on the Beautifil II before the contamination (green curve in Figure 4). Such large spectral features can be ascribed to a possible fluorescence signal coming from the organic residues.

Differently, the spectra taken at different points on the back side (Figure 6(b)) demonstrate that the contamination is less present, and the surface appears more uniform and similar to that tested before the contact with saliva (Figure 4).

**Figure 7:**
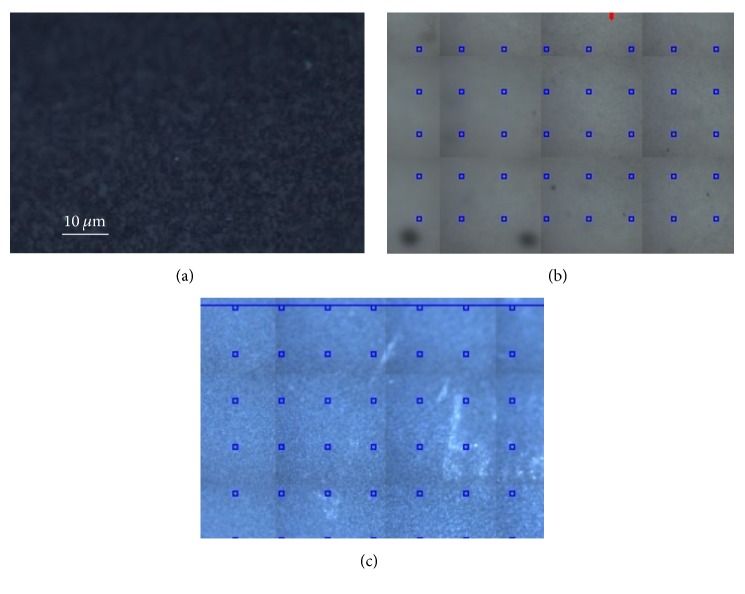
Typical optical images obtained on the Dyract with a 50x objective. (a) Before contamination; (b) after contamination, front side; (c) after contamination, back side.

**Figure 8:**
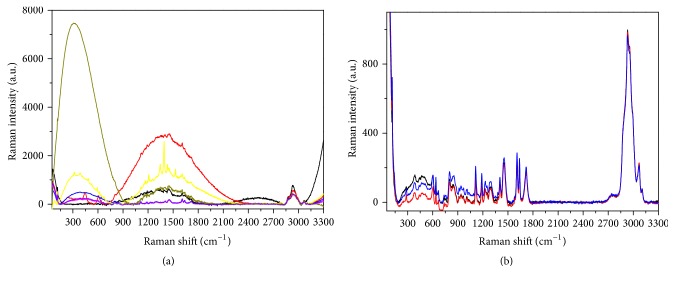
Typical Raman spectra collected on the Dyract after contamination. (a) Front side; (b) back side. In both cases the differently colored curves correspond to accumulated measurements taken at some representative points of the grids visible in Figures 7(b) and 7(c).

Similar results have been obtained on the Dyract: Figure 7 reports the optical images of the sample before (a) and after the contamination ((b) front side; (c) back side), while the Raman data are shown in Figure 8.

Also in this case, the back of the material is not dirty, while the spectrum of the front side is characterized by large bands related to organic residues.

**Figure 9:**
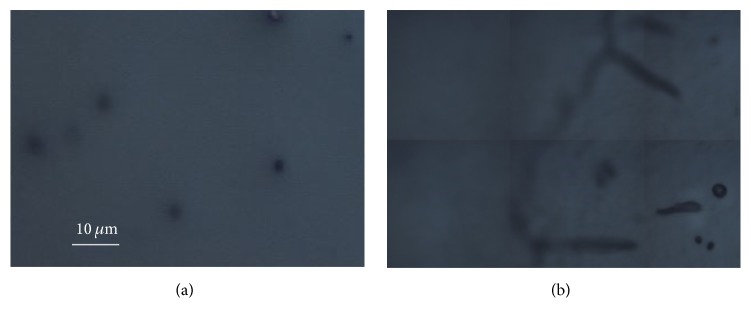
Typical optical images obtained on the Ketac with a 50x objective. (a) Before contamination; (b) after contamination, front side.

As regards the Ketac, the sample appears quite glossy and less flat than the others (it was not possible to realize a good optical image on the back side, so that Figure 9 just presents those realized on the front side before (a) and after (b) the contamination).

**Figure 10:**
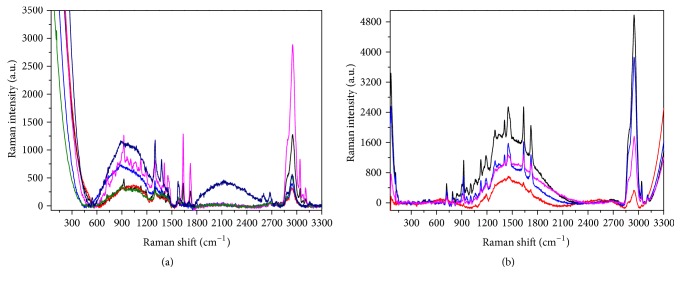
Typical Raman spectra collected on the Ketac after contamination. (a) Front side; (b) back side. In both cases the differently colored curves correspond to accumulated measurements taken at some representative points on the sample surface.

From the spectral point of view, after the contact with saliva, the front side of Ketac seems less altered (Figure 10(a) showing less intense fluorescence bands with respect to the other samples). However, by comparing the data of Figures 10(a) and 10(b), one can notice that the two sides have similar spectra, demonstrating that the contamination also involves the back side. The reason for this behavior could be in the different absorbing/slipping properties of the sample surface that reduce the permanence time of the saliva on the first side, allowing it to reach the back one.

**Figure 11:**
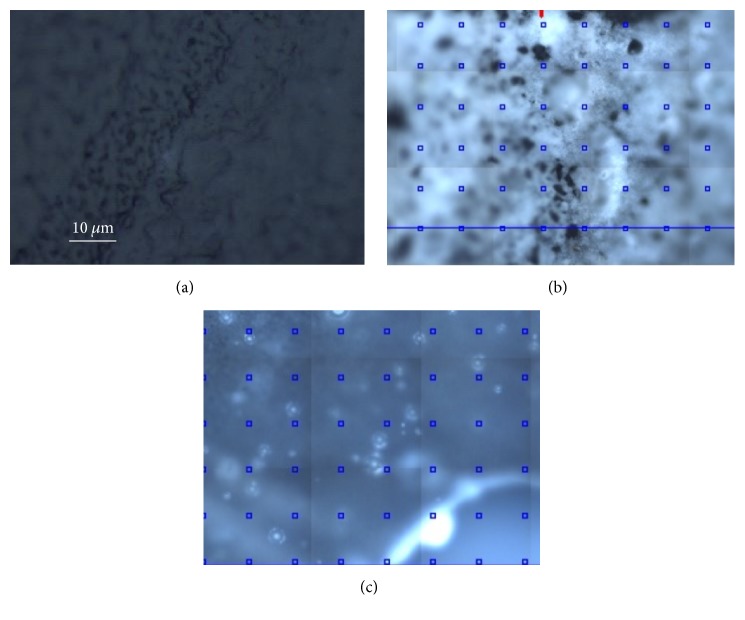
Typical optical images obtained on the Tetric with a 50x objective. (a) Before contamination; (b) after contamination, front side; (c) after contamination, back side.

**Figure 12:**
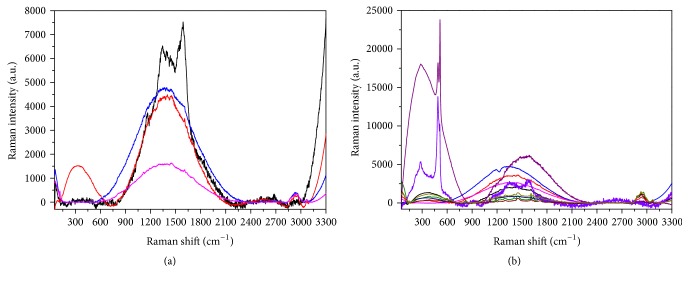
Typical Raman spectra collected on the Tetric after contamination. (a) Front side; (b) back side. In both cases the differently colored curves correspond to accumulated measurements taken at some representative points of the grids visible in Figures 11(b) and 11(c).

Finally, we have analyzed the surface properties of Tetric. Like in the case of Ketac, both the front and back side appear spotted and soiled, as it is possible to observe both in the optical images of Figure 11 and in the graphs of Figure 12.

However, the intensity of the spectral contributions ascribable to the contamination is more intense here than in Ketac, indicating a higher concentration of organic residues.

### 3.5. Ageing Test in Sugary Drink-Comparison

**Figure 13:**
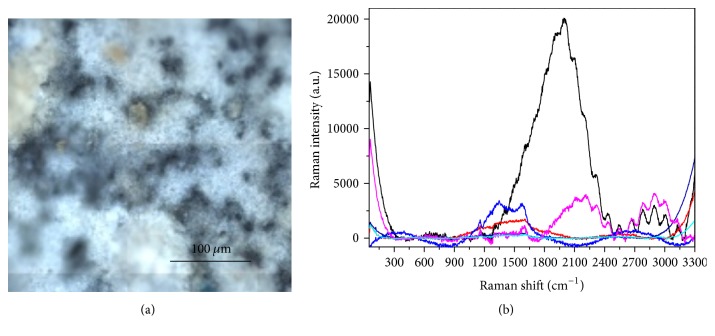
Analysis of the surface of Beautifil II after contamination with sugar-drink. (a) Optical image. (b) Raman spectra collected in different points.

The samples were mapped with the Raman microscope on multiple points, revealing an inhomogeneous surface with some contaminations resulting from organic residues, primarily sugars.  Figure 13(b) presents the spectra obtained on the surface of Beautifil II, showing fluorescent mixed contributions that are more intense on the areas that appear white in the optical image (Figure 13(a)).

**Figure 14:**
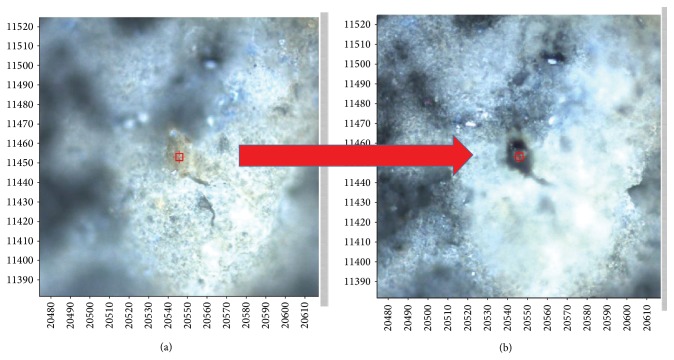
Optical images taken on the sample before (a) and after (b) the collection of a single spectrum centered on a “brown grain.”

Moreover, the collected spectra display clear sign of sample local heating, because the investigated areas are covered with organic or polymeric material that tends to burn during the measurement. In fact, these contaminants can be burnt by the laser source of the Raman apparatus even after an exposure of a few tens of seconds, thus leaving a typical dark imprint, as can be observed from the optical images reported in Figure 14 and taken on the sample before (a) and after (b) the collection of a single spectrum centered on a “brown grain.”

**Figure 15:**
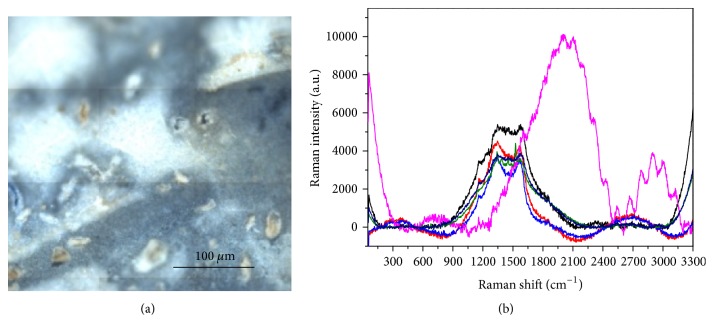
Analysis of the surface of Dyract after contamination with sugar-drink. (a) Optical image. (b) Raman spectra collected in different points.

Organic residues are observed on the surfaces of all four materials, this phenomenon being due to the interaction with the sugary drink, so that also all the spectra measured on Dyract and reported in Figure 15 present the same large bands due to both fluorescence and local heating already found on Beautifil II.

**Figure 16:**
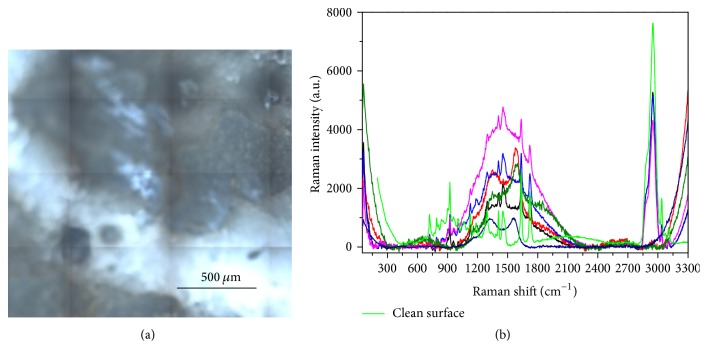
Analysis of the surface of Ketac after contamination with sugar-drink. (a) Optical image. (b) Raman spectra collected in different points. The spectrum of the clean surface (green curve) has been added for comparison.

A slightly different result has been obtained on Ketac, whose Raman data clearly show the spectral characteristics of the clean material under the usual overlapping large bands (see Figure 16 in which the spectrum of the clean surface has been added for comparison). This finding could indicate a smaller degree of contamination, with nonuniform coverage of the surface, in agreement with the results shown in Figure 10 for the case of the contact with saliva.

**Figure 17:**
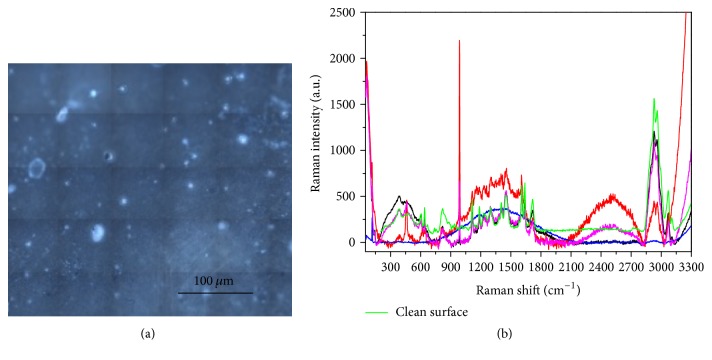
Analysis of the surface of Tetric after contamination with sugar-drink. (a) Optical image. (b) Raman spectra collected in different points. The spectrum of the clean surface (green curve) has been added for comparison.

Finally, in the case of Tetric, beside the presence of spectral signatures ascribable to fluorescent and heating/burning phenomena superposed to the spectrum of the clean sample, that indicates, again, an inhomogeneous coverage with contaminants, two novel peaks, a narrow one at about 898 cm^−1^ and a larger one at about 2450 cm^−1^, suggest a possible occurrence of some chemical interaction between the Tetric surface and the sugary drink, causing permanent alteration of the sample (Figure 17).

### 3.6. Weight Variation in Saliva

**Figure 18:**
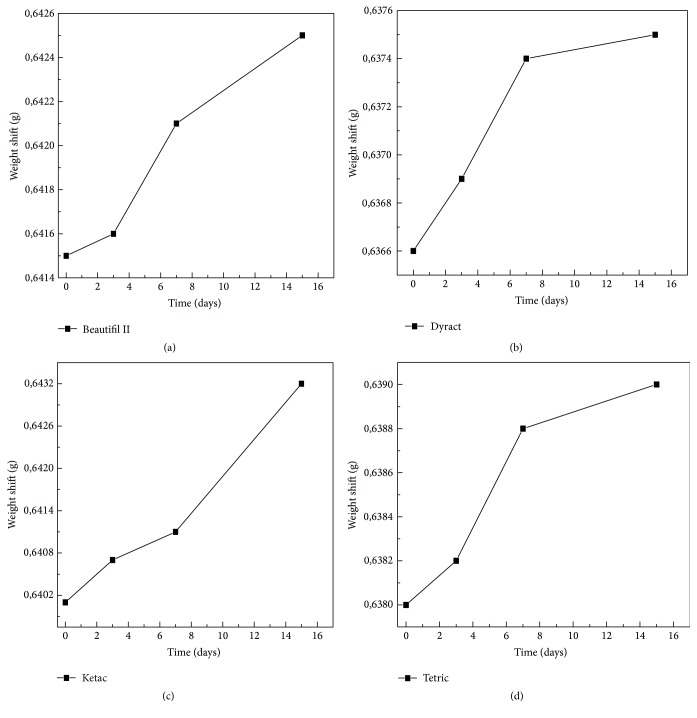
No weight decrease has been observed in the four materials in exam.

As can be seen from Figures 18(a), 18(b), 18(c), and 18(d), for all the four types of materials no weight decrease has been found due, for example, to possible loss of material, but a small maximum increase of 0.4% is observed, probably caused by organic compounds measured by the Raman.

The measures are therefore consistent (see Table 1).

**Figure 19:**
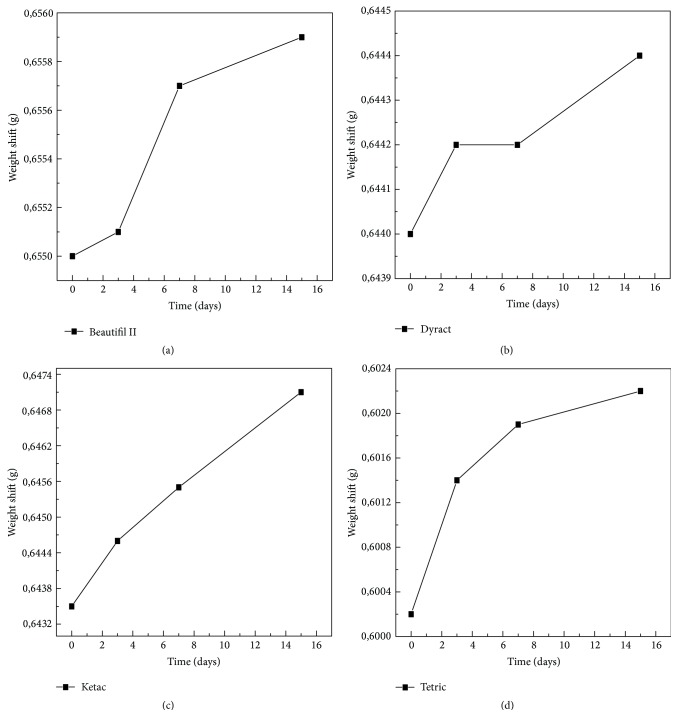
Changes in weight after ageing in the sugary drink.

The same goes for changes in weight after ageing in the sugary drink as depicted in Figures 19(a), 19(b), 19(c), and 19(d), while the numerical variation can be observed in Table 2.

## 4. Discussion

Giomers represent a new category of restoratives with promising clinical behavior and good mechanical stability [26].

Giomers have the fluoride release and recharge properties of glass ionomer cement. They are able to recharge fluoride when treated with fluoridated products, decrease acid production of cariogenic bacteria, and neutralize acid on contact and are capable of slow demineralization, while promoting remineralization of enamel demonstrates an antiplaque effect; many important studies were conducted on these fundamental properties [2, 27–38].

New, numerous, and specific clinical applications are now feasible thanks to the aesthetic potential offered by the PRG technology of Giomers [6, 14–18, 39–45].

As regards the mechanical characteristics in the scientific literature few studies are actually present on Giomer materials [2, 7–13, 19, 20, 22–24, 26, 42].

In this in vitro study, a comparative analysis was presented to investigate the different mechanical and morphological properties and ageing of four classes of restorative materials.

As before analysis, the hardness of all materials was studied through the indentation strength tests technique.

The results obtained have shown very similar hardness for composite resin (Tetric) and Compomer (Dyract) while that of Giomer (Beautifil II) is almost double.

This is in agreement with a study of Yap et al. in which the comparison of hardness of six restorative materials (an Ormocer, a Giomer, a Compomer, a minifill composite, resin-modified cement, and highly viscous glass ionomer cement) led to the conclusion that the ranking of mechanical properties was generally similar, and no significant change in hardness was observed for all materials in exam with thermocycling, although the Giomer is significantly harder [39].

Also Ilie and Fleming, investigated on micromechanical properties (Vickers hardness/HV; depth of cure/DOC; indentation modulus/E) of Giomer materials ascertaining that they are higher compared with the conventional resin-based composite materials [26].

This physical capability could be as much tied to the PRG filler as the chemical nature of the Giomer product having cross-linked polymer matrices so the hardness such as the compressive strength and toughness of the material also seems to be higher than the gel network formed by acid-base reaction in glass ionomers [22].

As regards the glass ionomer cement (Ketac) the indentation strength tests performed in the present study reveal an even higher hardness, although there are major fluctuations probably due to a not complete homogeneity of the material. In fact, this suggests the presence of basically two zones with different hardness.

In a recent study it was reported that no degradation with ageing (in distilled water to 37°C) is identified in the Giomer restorative. The gradual ascending transition in micromechanical properties from dentin through dental bioactive restoratives identified in both restoratives might have a positive effect on the bond quality [25].

An in vitro study, to assess hardness and elastic modulus of three restorative materials (Beautifil II, Gradia Direct X, and Tetric Evoceram) following ageing in deionized water (pH 6.5) and lactic acid (pH 4.0), analyzed the fluoride release and recharge of these fluoride-containing resin composites comparing them to glass ionomer cement (Fuji IX Extra) taken as a reference material. With respect to all the examined materials, Giomer possesses the highest properties of fluoride release and recharge. From this result it is possible to demonstrate the particular behavior of this composite material. The mechanical properties of Giomer did not diminish with ageing and fluoride release [46].

The spectrophotometric analysis carried out on the specimens showed no particular differences, especially in the visible range, where there are no abnormal absorption for the materials.

Even the Raman analysis on the samples that did not undergo the ageing process was similar between the different components in question.

In literatures, it is reported that significant changes of surface can take place on both tooth enamel and dental restorative materials when exposed to low pH drinks such as carbonated drinks (cola) and fruit juice (orange fruit juice, apple cider), coffee, and beer for a prolonged period. They consist in microhardness and surface characteristic changes principally [47, 48].

Giomer restoratives are significantly roughened by citric acid [10]. The erosive potential is calculated by surface topography observation through scanning electron microscope and by measuring the surface roughness value [11]. Compared to Compomer, Giomer shows less surface roughness and better color stability than resin-modified glass ionomer cement [9, 12].

Instead the study of ageing in human saliva, performed in this work, showed in general a surface contamination of inhomogeneous materials. In particular, the bands of Raman spectra suggest the presence of organic residues of different types of proteins. Moreover the contamination on the two sides of the specimens is also different in the majority of the cases. A possible cause of this behavior is the presence of mucus deposited on only one side of the samples, due to the separation of mucus from the liquid saliva during the days inside the test vial. In the case of ageing in the sugary drink, three materials (Beautifil II, Dyract, and Ketac) show organic contamination linked to the presence of sugars on both sides, while Tetric shows the presence of peaks related to a partial modification of the surface chemical bonds to be attributed to long-term interaction. These results suggest that Giomer is not particularly damaged by the usage of sugary drink even if the surface can appear dirtier. Finally the measures of weight changes for the aged samples both in saliva and in the beverage showed no decreases and then release of material, but have confirmed the Raman measurements, showing a small positive variation due to the presence of residues on the surface. These changes are at most of the order of 5 per thousand.

## 5. Conclusions

Not surprisingly, given their composition, Giomers show very similar behavior to the other restorative materials investigated in this study. It is important remarking that Giomer demonstrated a resulting hardness 2 times higher than Compomer and composite resin despite the release and recharge capability of the material.

It is doubtful whether the inclusion of prepared glass ionomers makes any significant difference to these materials, and they are not fundamentally new materials, but a very slightly modified version of well-established materials.

Aesthetic dentistry is considered a form of art, which requires vision to express possibilities and skill to meet the demands of the patient. Perfection in aesthetic restorations cannot be achieved without unstinted dedication, commitment, and passion toward the profession. We should also keep abreast of new trends and continue to enhance knowledge of treatment options in terms of principles, procedures, materials, and the latest techniques.

## Figures and Tables

**Table 1:** 

Material	Samples	Variation (%)
BEA II	4	0,156
Dyract	4	0,141
Ketac	4	0,484
Tetric	4	0,156

**Table 2:** 

Material	Samples	Variation (%)
BEA II	4	0,137
Dyract	4	0,047
Ketac	4	0,550
Tetric	4	0,333
